# The evolution of pulmonary pathology in fatal COVID-19 disease: an autopsy study with clinical correlation

**DOI:** 10.1007/s00428-020-02881-x

**Published:** 2020-06-30

**Authors:** Hans Bösmüller, Selina Traxler, Michael Bitzer, Helene Häberle, Wolfgang Raiser, Dominik Nann, Leonie Frauenfeld, Antonio Vogelsberg, Karin Klingel, Falko Fend

**Affiliations:** 1 Department of Pathology and Neuropathology, University Hospital Tübingen and Eberhard Karls University Tübingen, Liebermeisterstraße 8, 72076 Tübingen, Germany; 2 Department of Internal Medicine I, University Hospital Tübingen, Tübingen, Germany; 3 Department of Anaesthesiology, University Hospital Tübingen, Tübingen, Germany; 4 Office for General Medicine, Tübingen, Germany

**Keywords:** COVID-19, Autopsy, Lung, Capillaritis, Microthrombosis

## Abstract

The pandemia of coronavirus disease 2019 (COVID-19) has caused more than 355,000 confirmed deaths worldwide. However, publications on postmortem findings are scarce. We present the pulmonary findings in four cases of fatal COVID-19 with a spectrum of lung pathology reflecting disease course and duration, invasive therapies, and laboratory features. Early disease is characterized by neutrophilic, exudative capillaritis with microthrombosis and high levels of IL-1beta and IL-6. Later stages are associated with diffuse alveolar damage and ongoing intravascular thrombosis in small to medium-sized pulmonary vessels, occasionally with areas of infarction equivalents, accompanied by laboratory features of disseminated intravascular coagulation. In late stages, organizing pneumonia with extensive intra-alveolar proliferation of fibroblasts and marked metaplasia of alveolar epithelium can be observed. Viral RNA is encountered in the lung, with virus particles in endothelial cells and pneumocytes. In many patients, multi-organ failure with severe liver damage sets in finally, possibly as consequence of an early-onset pro-inflammatory cytokine storm and/or thrombotic microangiopathy.

**Electronic supplementary material:**

The online version of this article (10.1007/s00428-020-02881-x) contains supplementary material, which is available to authorized users.

## Introduction

Since the outbreak of coronavirus disease 2019 (COVID-19) caused by the novel coronavirus SARS-CoV-2 in December 2019, almost 5.6 million people have been infected during the course of the pandemia so far, and more than 355,000 succumbed to the disease [1]. Severe pneumonitis advancing to acute respiratory distress syndrome (ARDS), frequently associated with multi-organ failure, has been observed as the main clinical feature in fatal cases, with a case fatality rate of 1–4% [2, 3]. Despite the massive scale of the pandemia, published data on autopsy findings are limited [4–8]. In addition to diffuse alveolar damage, severe endothelial injury, disseminated intravascular coagulation, and a pro-inflammatory cytokine storm are considered main pathogenic factors in severe disease [4, 9]. Early autopsy studies documented respiratory failure due to ARDS as predominant cause of death, frequently accompanied by capillary microthrombosis, superimposed bronchopneumonia, pulmonary thromboembolism, and signs of multi-organ failure with shock organs [8]. Most but not all patients with fatal outcome are elderly and show a variety of risk factors and comorbidities, including current smoking, chronic obstructive pulmonary disease, congestive heart failure, coronary artery disease, and diabetes mellitus [3, 10, 11]. Since the beginning of the epidemic in Germany, we have performed autopsies of confirmed COVID-19 cases at the Institute of Pathology, University Hospital Tuebingen, Germany. In the four cases presented here, we focus on the correlation between the autopsy findings, clinical course, and laboratory findings and demonstrate that the range of pulmonary pathology encountered reflects the duration of the disease, underlying pathophysiological mechanisms, type and intensity of treatment, and preexisting conditions.

## Methods

### Autopsy

For all deceased patients, informed consent had been obtained from the next of kin, and autopsies were performed following proposed guidelines for hazard group 3 pathogens, concerning autopsy practice and personal protective equipment [7, 12]. In order to minimize risk, aerosol formation was avoided as much as possible, and craniotomy and dissection of the central nervous system were not performed. Both lungs were submerged in and filled with buffered formalin. All major parenchymal organs were extensively sampled for histology. In addition, specimens of both lungs, heart tissue, skeletal muscle, and liver were obtained for virological studies and electron microscopy.

### Histology and Immunostaining

Routine histological stains were performed on formalin-fixed and paraffin-embedded samples following standard protocols. Immunohistochemistry was performed on an automated immunostainer (Ventana Benchmark Ultra, Roche Diagnostics, Mannheim, Germany).

### Electron microscopy

For electron microscopy, tissues were fixed in 2.5% glutaraldehyde (Paesel-Lorei, Frankfurt, Germany) buffered in 0.1 M cacodylate buffer (pH 7.4). Tissues were postfixed in 1% OsO_4_ in 0.1 M cacodylate buffer and then dehydrated in an ethanol series (50, 70, 96, 100%). The 70% ethanol was saturated with uranyl acetate for contrast enhancement. The specimens were embedded in Araldite (Serva, Heidelberg, Germany). Ultrathin sections were contrasted with lead citrate and analyzed and documented with an EM10A electron microscope (Carl Zeiss, Oberkochen, Germany).

### RNA studies for SARS-Cov-2 RNA and IL-1beta and IL-6 mRNA detection

The details for the quantitative RT-PCR studies for the detection of SARS-CoV-2 and IL-1beta and IL-6 mRNAs are described in the supplement.

## Results

### Clinical histories

**Fig. 1:**
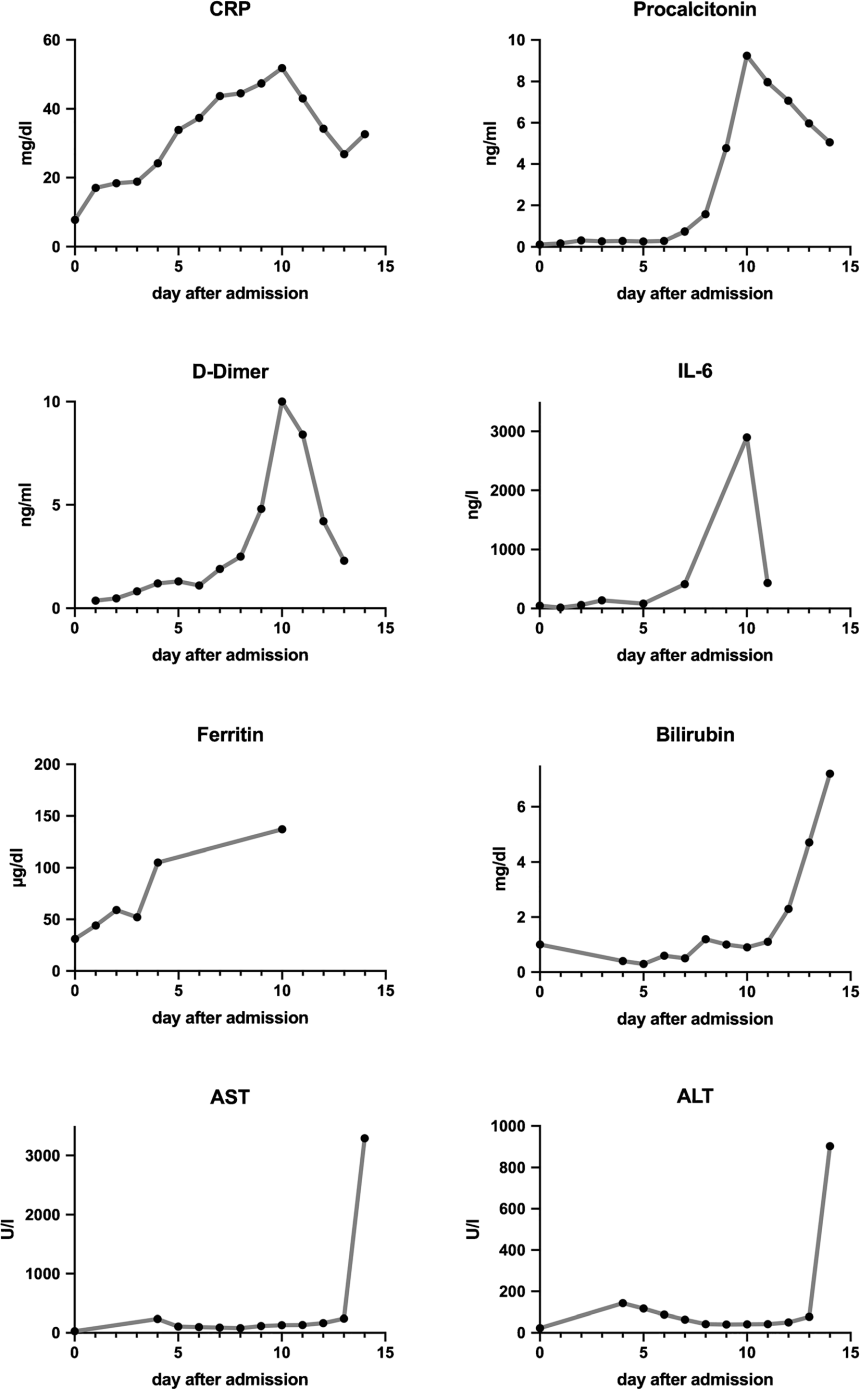
Laboratory data patient 3

Details on the patients are listed in Table 1. Selected laboratory parameters during the disease course are shown in Fig. 1 and supplementary Fig. 1. All patients had tested positive for SARS-CoV-2 by qRT-PCR at diagnosis in a throat swab.

**Table 1:** Clinical data of the patients

Age	Sex	Days from admission until death	Days in ICU	Days on dialysis	Comorbidities	Clinical cause of death
78	w	1 (home care)	–	–	Cardiac pacemaker, obesity	Pneumonia
79	m	9	8	3	Coronary heart disease, diabetes type 2, obesity, hypertension, Parkinson’s syndrome	ARDS, liver failure, shock
72	m	16	11	6	Coronary heart disease, Merkel cell carcinoma under adjuvant radiotherapy, obesity, polymyalgia rheumatica	ARDS, liver failure, shock
59	M	35	35 (24 on ECMO)	24	Hypertension, intrinsic asthma	ARDS, multi-organ failure

*ICU* intensive care unit, *ARDS* acute respiratory distress syndrome, *ECMO* extracorporeal membrane oxygenation

Patient 1 was a 78-year-old obese female (BMI 35.2 kg/m^2^) with hypertension and an atrioventricular block treated with a permanent dual chamber pacemaker. The patient died at home, after experiencing a 12-h period of fever with cough and vomiting according to her relatives. A throat swab performed few hours before death tested positive for SARS-CoV-2 by qRT-PCR. No other laboratory data were available. Clinical autopsy was requested by her relatives and was undertaken 48 h after death.

Patient 2 was a 78-year-old male patient with history of general weakness for 3 weeks, fever, and dry cough with worsening symptoms during the last 3 days before admission. The past medical history included coronary artery disease, hypertension, diabetes, and Parkinson’s disease (BMI 28.4 kg/m^2^). At admission, his blood pressure was 167/74 mmHg, body temperature 36.2 °C, respiratory rate 20/min, and SpO_2_ 92% breathing ambient air. Laboratory results showed lymphopenia of 940/μl (normal 1.100–3.200), increased D-dimer with 0.87 μg/ml (< 0.5), fibrinogen 574 mg/dl (170–410), C-reactive protein (CRP) 29.5 mg/dl (≤ 0.5), IL-6 100 ng/l (0–4), LDH with 479 U/l (≤ 250) creatine kinase 280 U/l (≤ 190), and ferritin 113 μg/dl (3–30) (see also supplementary Fig. 1). He was transferred to the ICU the next day with worsening respiratory symptoms, including a respiratory rate of 38/min and SpO_2_ 91% under O_2_ 6 l/min. He was intubated due to respiratory failure. Several blood cultures for bacteria or fungi remained negative. Overall organ function improved in the next 2 days. At that point in time, IL-6 and D-dimer concentrations peaked at 3.300 ng/l and 19 μg/ml FEU respectively, whereas thrombocytopenia worsened despite anticoagulation. Within the following 24 h, the patient developed multi-organ failure and vasoplegic shock. Leukocytes, procalcitonin, and CRP concentrations were increasing continuously despite lack of evidence for superinfection, whereas D-dimer and IL-6 concentrations dropped (see supplementary Fig. 1). The patient died 4 days after peak of D-dimer concentration due to vasoplegic shock and liver failure despite vasopressor and organ replacement therapy. The autopsy was performed within 24 h after death.

Patient 3 was a 72-year-old male admitted with syncope, fever, cough, and emesis. The past medical history included coronary artery disease, hypertension, currently untreated polymyalgia rheumatica, and a history of Merkel cell carcinoma with ongoing local adjuvant radiotherapy (BMI 22.3 kg/m^2^). At admission, his blood pressure was 95/60 mmHg, body temperature 38.3 °C, respiratory rate 10/min, and SpO_2_ 95% breathing ambient air. Laboratory results showed lymphopenia with 440/μl, increased CRP 7.7 mg/dl (≤ 0.5) and IL-6 27 ng/l (0–4), and normal values for LDH, ALT/AST, bilirubin, and D-dimer (see Fig. 1). He was transferred to the ICU 4 days later with worsening respiratory symptoms and recurrent fever episodes and was intubated. Several blood cultures for bacteria remained negative. Oxygenation improved until day 6 day after ICU admission, when acute hypercapnia became apparent. Marked leukocytosis and increase in D-dimer, C-reactive protein, IL-6, and procalcitonin levels developed. Pulmonary superinfection with *Klebsiella oxytoca* was diagnosed, and meropenem therapy was started. The patient subsequently developed renal and liver failure, and IL-6 and D-dimer levels decreased. Renal replacement therapy was initiated, but he expired 10 days after admission to ICU due to liver failure. The autopsy was performed within 24 h after death.

Patient 4 was an obese 59-year-old male with a history of intrinsic asthma and hypertension (BMI 35.8 kg/m^2^). He complained about respiratory symptoms for 2 weeks prior to admission. He was admitted to the ICU of a peripheral hospital and transferred to our hospital 6 days later for his respiratory failure and started on extracorporeal membrane oxygenation (ECMO). In addition, he underwent dialysis. During the course of his disease, he experienced two episodes with marked increase in D-dimer levels, the second 4 days before his death. He expired due to terminal ARDS and multi-organ failure 6 weeks since the beginning of his symptoms. The autopsy was performed within 24 h after death.

#### Autopsy findings

Details on the organ weights (formalin-fixed) are listed in supplementary Table 1.

##### Patient 1

Grossly, lungs showed significant pulmonary edema with slightly increased consistency of lower lobes. No focal changes were observed on cut surfaces of the lung. The heart showed an increased weight of 520 g and biventricular dilation. Sclerosis of the coronary arteries or signs of ischemia in the hyperplastic myocardium were not observed. All other organs were normal upon gross inspection.

**Fig. 2:**
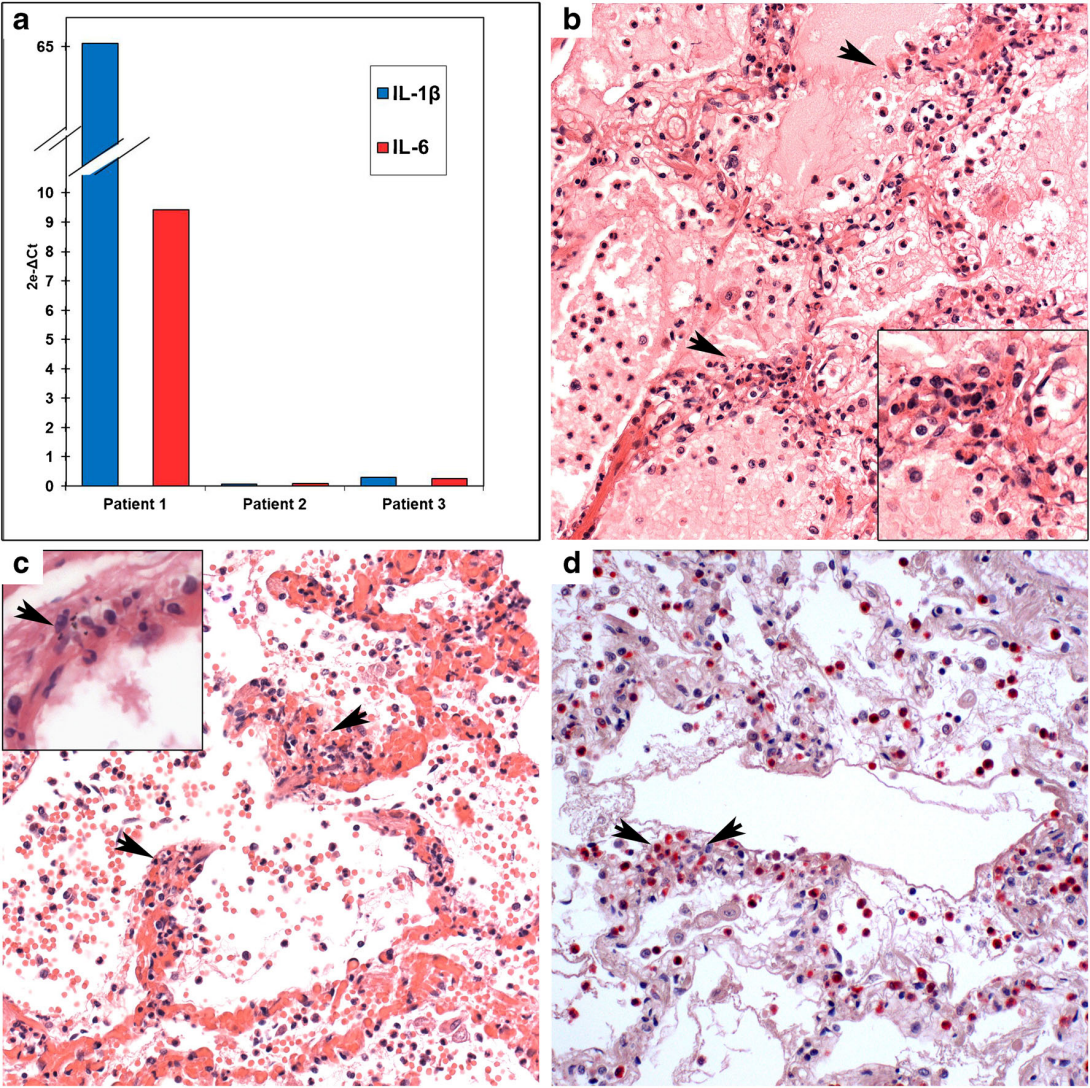
Patient 1. **a** Significant increase of IL-1beta and IL-6 mRNA in lung tissue of patient 1 as compared with patients 2 and 3. **b** Neutrophilic septal capillaritis (arrowheads) with associated intra-alveolar edema. H&E (× 200). The insert shows nuclear dust in higher magnification. **c** Neutrophilic capillaritis associated with microthrombosis (arrowheads) and intra-alveolar hemorrhage. H&E (× 200). The insert shows endothelial necrosis with nuclear dust (arrow) and transmigration of neutrophils (× 400). **d** NASD chloroacetate esterase histochemistry highlights intra- und perivascular accumulation of neutrophils (granulocytes appear red) (× 200)

Histologically, in addition to marked generalized pulmonary edema, the lower lobes showed florid capillary endotheliitis with increased neutrophils, formation of microthrombi in alveolar capillaries, and small pulmonary vessels, including septal veins. In addition, focal inflammatory exudate with neutrophils and sparse hyaline membranes with incipient organizing changes but without hyperplasia of alveolar epithelium were observed (Fig. 2). The liver showed moderate acute congestion and activation of Kupffer cells but lacked inflammatory infiltrates. qRT-PCR of cytokines in lung tissue revealed a massive increase of IL-1beta and IL-6 mRNA. Early pneumonitis with thrombotic microangiopathy resulting in inflammation-associated pulmonary edema and acute cardiac failure was considered the likely cause of death.

##### Patient 2

**Fig. 3:**
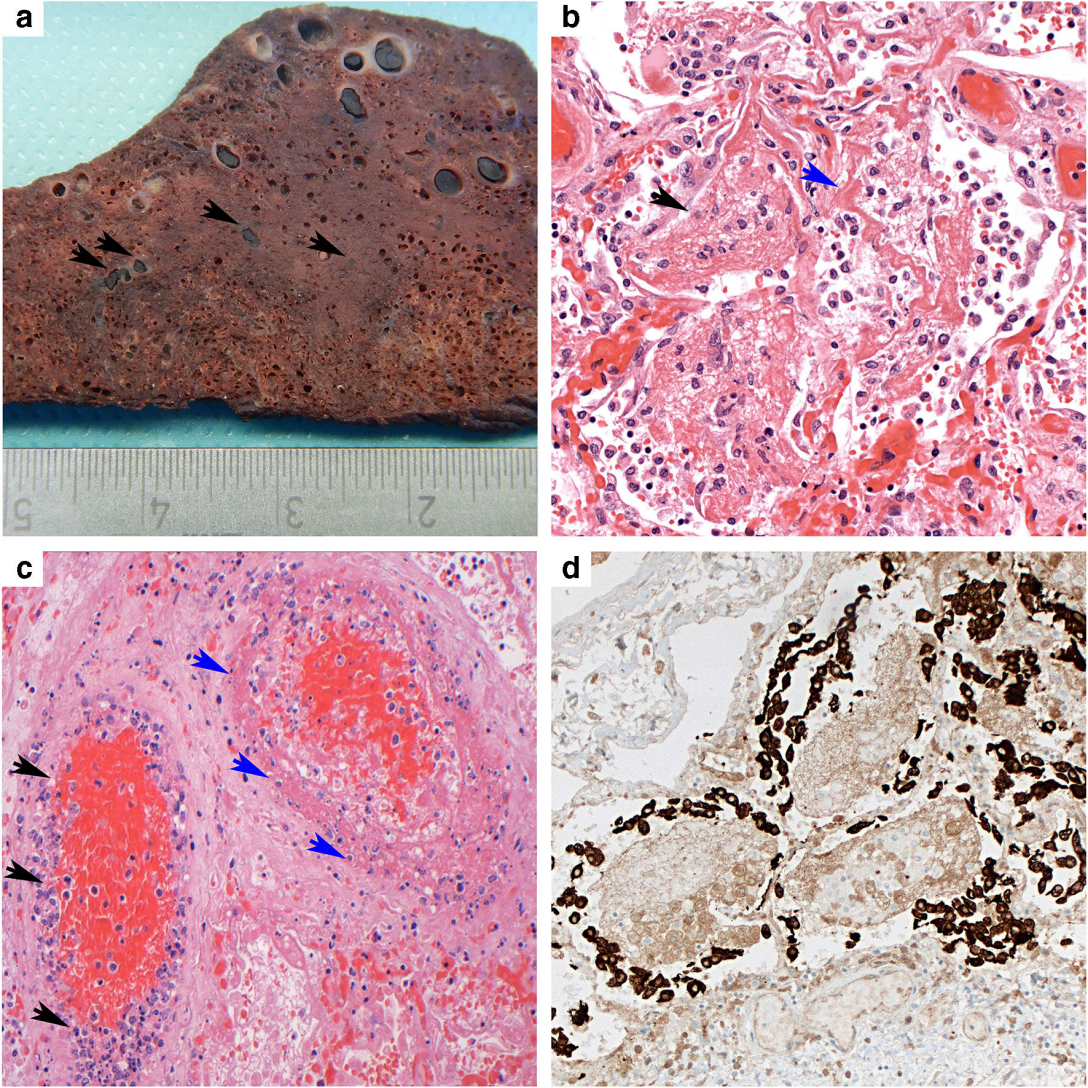
Patient 2. **a** Gross image of cut surface of the lower pulmonary lobe with multiple thrombi in small vessels, focally accompanied by perifocal hemorrhage (arrows) and consolidation and discoloration of the parenchyma. **b** The lower lobes show advanced diffuse alveolar damage with fibrinous exudate (black arrowheads), hyaline membranes (blue arrowheads), incipient hyperplasia of alveolar epithelium, and increased desquamation of macrophages. H&E (× 200). **c** Thrombosis of two medium-sized pulmonary blood vessels with marked endotheliitis (black arrowheads), fibrinoid necrosis of the vessel wall (blue arrowheads), and thrombi in engorged surrounding capillaries. H&E (× 200). **d** Massive hyperplasia of type 2 pneumocytes surrounding the fibrinous exudate with macrophages. Pan-cytokeratin (AE1/3) immunohistochemical stain (× 200)

Gross inspection showed marked pulmonary edema, consolidation of both lower lobes and macroscopically visible thrombi mainly in small to medium-sized pulmonary vessels, both arteries and veins, in part accompanied by areas of fresh infarction (Fig. 3a, c). Moderate hepatosplenomegaly was observed, and the other organs were inconspicuous.

**Fig. 4:**
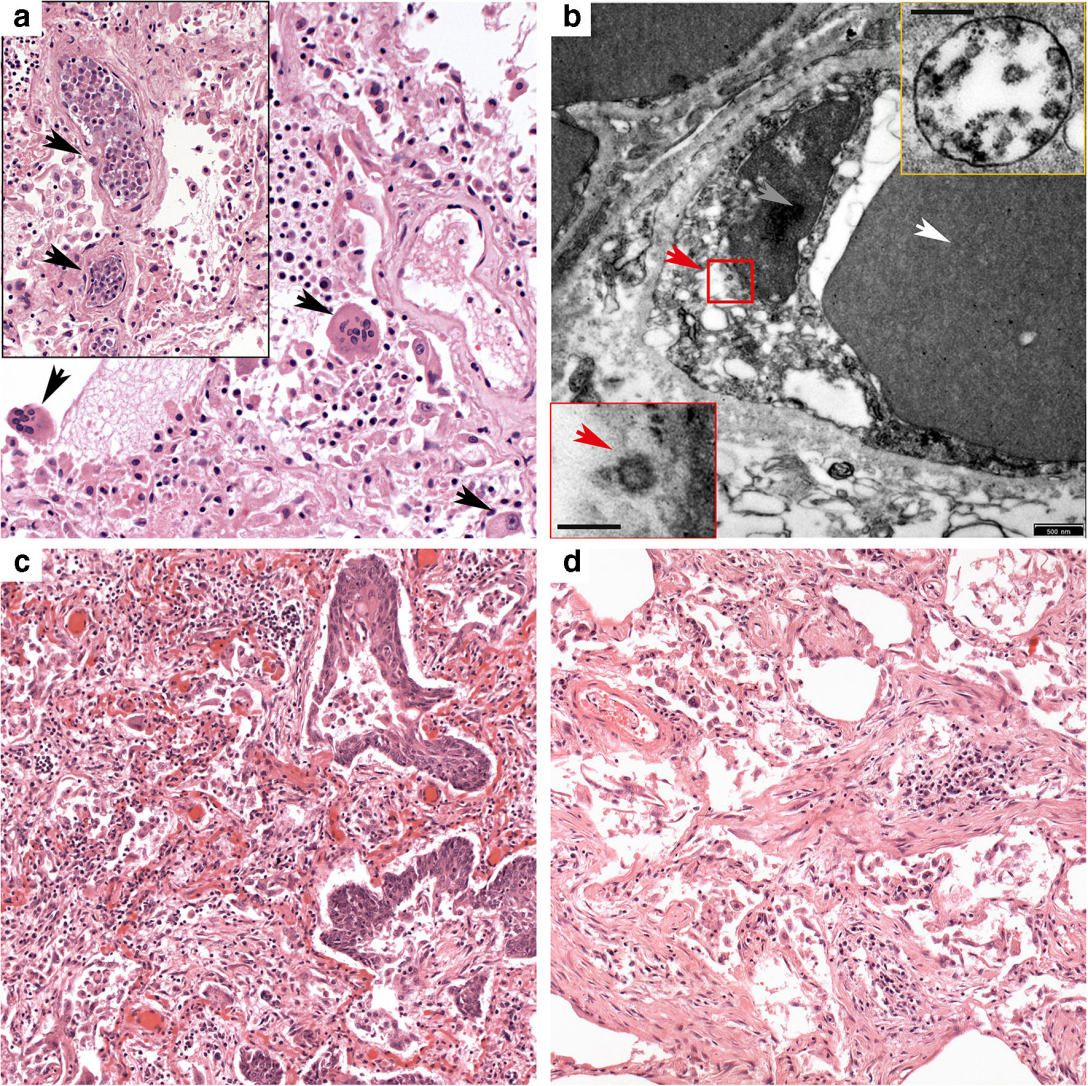
Patients 3 and 4. Patient 3. **a** Lower lobe with diffuse alveolar damage with multinuclear alveolar macrophages (arrows) and striking leukostasis (insert), both H&E (× 200). **b** Virus particles (red arrowhead) are often found in endothelial cells (white arrowhead, erythrocyte; gray arrowhead, nucleus of endothelial cell); insert bottom, bar scale 100 nm. The majority of viruses are located in membrane-bound compartments, likely representing endosomes, insert top (bar scale 200 nm). Patient 4. **c** The lung shows features of prolonged ARDS with extensive epithelial hyperplasia and focal squamous metaplasia. H&E (× 100). **d** Typical changes of organizing pneumonia with plugs of loose connective tissue with concentrically arranged fibroblasts and central accumulation of inflammatory cells filling alveolar spaces. H&E (× 100)

The lung revealed diffuse alveolar damage with extensive intra-alveolar fibrin deposits with formation of hyaline membranes, marked hyperplasia and desquamation of alveolar epithelium, and accumulation of macrophages with frequent multinuclear giant cells (Figs. 3b, d and 4a). Especially the lower lobes showed areas of organized diffuse alveolar damage (DAD) with proliferation of fibroblasts and early collagen fiber deposits within the intra-alveolar exudate. A striking finding was a focally massive accumulation of leukocytes in medium-sized vessels, but florid neutrophilic capillaritis was absent. The liver showed significant activation of macrophages with signs of hemophagocytosis, but no necrosis or inflammatory infiltrates. Electron microscopy demonstrated viral particles in pulmonary endothelial cells and also in pneumocytes type 1 (Fig. 4b and supplementary Fig. 2). IL-1beta and IL-6 mRNA were not increased in lung tissue.

##### Patient 3

Gross and microscopic pulmonary changes were similar to patient 2, with macroscopically identifiable thrombi in pulmonary vessels and consolidation of both lower lobes. The liver showed enlargement with yellowish tan surface, and splenomegaly was present. Pulmonary histology showed changes of advanced diffuse alveolar damage, with extensive hyaline membranes and intra-alveolar macrophage accumulations with multiple giant cells and pronounced, in part atypical hyperplasia of alveolar epithelium with focal squamous metaplasia and areas of organizing pneumonia (Fig. 4) Neutrophils were infrequent, arguing against significant bacterial superinfection. Viral particles were observed in endothelial cells of lung capillaries and also in vacuoles within the interstitial space (Fig. 4b). IL-1beta and IL-6 mRNA were not increased in lung tissue.

##### Patient 4

Demonstrated lungs with massively increased weight (left, 828 g; right, 1032 g) and significant consolidation in both upper and lower lobes. In addition to cardiomegaly with a heart weight of 590 g and signs of liver damage, he revealed intestinal mucositis and hemorrhage, probably responsible for the second increase in D-dimers.

Histologically, the lungs showed the aspect of long-standing ARDS in organizing stage with extensive fibrinous exudates, diffuse thickening of alveolar septae, massive hyperplasia of alveolar and bronchial epithelium with focal squamous metaplasia, and typical concentrically layered plugs of loose connective tissue with central aggregates of inflammatory cells (Fig. 4c, d).

#### Detection of viral RNA

Significant levels of SARS-CoV-2 RNA were detected in the lungs of all patients by qRT-PCR, but not in the livers and hearts. Details are summarized in supplementary Table 2.

## Discussion

Autopsies are an important tool to investigate the pathogenetic mechanisms of severe SARS-CoV-2 infection by demonstrating morphological changes and other important features such as infected cell types and viral load in organs normally not accessible in severely ill patients. The four cases presented here highlight a range of pulmonary alterations with variable severity, which reflect different stages of disease evolution. Patients 2, 3, and 4 demonstrated extensive, advanced pulmonary changes typical for ARDS, leaving no doubt about the severity of lung damage, accompanied by advanced organizing changes in patient four, reflecting the prolonged clinical course. In contrast, patient 1 showed early changes with striking increases of IL1-beta and IL-6 mRNA, neutrophilic capillaritis, and capillary microthrombosis but relatively little parenchymal inflammation. Based on conventional criteria, respiratory insufficiency therefore might be considered unlikely direct cause of death, but this case and recently published autopsy data indicate that pulmonary microvascular changes are an important and distinguishing feature of COVID-19 and may contribute to hypoxemia and acute cardiac insufficiency. It is important to distinguish these findings from mere stress-related neutrophilia, which lacks microthrombus formation and presence of widespread, in part directly virus-induced endothelial damage [13]. Irrespective of the severity of pulmonary changes, however, all 4 patients showed SARS-CoV-2 RNA in lung tissues but failed to show detectable levels of viral RNA in other organs studied. The entry of SARS-CoV-2 into cells of the host is mediated by angiotensin-converting enzyme 2 (ACE2), a membrane bound metallopeptidase with broad mRNA expression in human tissues, with high levels of protein detectable on alveolar epithelial cells, intestinal epithelium, and both arterial and venous endothelial cells [14, 15] and the cellular protease TMPRSS2 [16]. High levels of viral RNA in pulmonary tissues in our cases, together with the histological findings, indicate that direct virus-induced damage is centered on the lung. Of interest, cytokine mRNA levels were high in the lung of patient 1 with very short disease course, whereas no significant levels were detected in patients 2 and 3, congruent with a drop in serum IL-6 levels before death. This could indicate that the massive secretion of cytokines occurs early in severe cases and might be a contributing factor to rapid disease evolution. The multi-organ failure observed in many patients is considered the result of a pro-inflammatory cytokine storm, including IL1β, IL2, IL6, IL7, IL8, IL10, IL17, and interferon gamma, probably accompanied by diffuse macrophage activation, as also evidenced by the presence of hemophagocytosis in liver tissue of our patients [9].

A common feature of severe COVID-19 is massive hypercoagulability with prominent elevation of D-dimer and fibrin/fibrin degradation products as in patients 2 and 3 [17–19]. This profound coagulopathy is considered responsible for the high incidence of thrombotic events and may be one reason for the high mortality in patients with cardiovascular risk factors and evidence of myocardial damage. Our autopsy findings support this contention, since endotheliitis with microthrombus formation and involvement of medium-sized vessels leading to pulmonary infarcts were prominent; however, obvious myocardial damage or lymphocytic inflammation was lacking. The neutrophilic capillaritis in patient 1 with short disease course indicates that severe endothelial damage due to viral infection, cytokines and induction of neutrophil extracellular traps (NETs) with endothelial apoptosis, and breakdown of the epithelial-endothelial barrier may be an early event in fatal cases [4, 6]. The massive vascular inflammation occasionally also observed in medium-sized vessels as shown in Fig. 3 is unusual for viral pneumonia without bacterial superinfection and may be a discerning feature of severe COVID-19. The hypercoagulability may also contribute to the observed mortality by triggering cardiovascular events before the development of severe pulmonary disease [11, 20, 21]. The frequent occurrence of pulmonary thromboembolism in fatal COVID-19 has recently been documented in an autopsy study [7, 8]. We assume that the combination of NET formation with their known pro-thrombogenic features, cytokine priming, and direct viral cytopathic effect on endothelia can rapidly induce disseminated intravascular coagulation [17, 22, 23].

In terms of risk factors and comorbidities, our four patients reflect the risk profiles observed during the pandemia [3, 11, 20]. Obesity, diabetes, hypertension, and pulmonary and cardiovascular disease are highly prevalent in patients with severe or fatal SARS-CoV-2 infection [3, 10, 11, 20]. The laboratory findings observed in patients 2 and 3 reflect common risk factors of fatal outcome, namely, lymphopenia; increased D-dimers; evidence of massive systemic inflammation including high levels of CRP, procalcitonin, and IL-6 during acute disease; and in the final stages massive ALT/AST elevation [1, 3, 4, 7, 11, 20]. The liver damage observed in our patients is likely not due to local inflammation or direct cytopathic effects of SARS-CoV-2, given the absence of viral RNA, but probably is a combination of cytokine-induced, metabolic, and ultimately hypoxic damage, as evidenced by massive centroacinar necrosis in patient 3 [24].

## Conclusion

Clinical autopsies promote our understanding of COVID-19 pathogenesis through assessment of tissue damage, virus distribution, and causes of death. In addition to severe pulmonary disease, disseminated coagulation and thrombus formation triggered by multifactorial endothelial damage are frequent events in fatal SARS-CoV-2 infection.

## Electronic supplementary material

ESM 1 (DOCX 987 kb)

## Data Availability

All data and material available.
