# Supplementary material for: The evolution of pulmonary pathology in fatal COVID-19 disease: an autopsy study with clinical correlation
Source: Virchows Arch. 2020 Jun 30;477(3):349–57. doi: 10.1007/s00428-020-02881-x (PMC7324489; doi:10.1007/s00428-020-02881-x)
Supplement: Supplementary file 1 — (DOCX 987 kb) [file 428_2020_2881_MOESM1_ESM.docx]

| *RNA studies for SARS-Cov-2 RNA and IL-1beta and IL-6 mRNA detection*  For RNA isolation, native tissue samples obtained during autopsy were placed in 500 μl peqGOLD TriFast RNA/DNA/Protein Isolation Reagent (peqLab Biotechnologie Erlangen, Germany). Tissue samples were mechanically shredded. After adding chloroform and 10 min of incubation at room temperature, samples were centrifuged and the aqueous supernatant, containing the RNA, was precipitated by adding 250 μl isopropanol. The RNA precipitate was washed with 75% ethanol and dried. The pellet was resuspended in 25 μl RNase free water.  Real-time quantitative RT-PCR was done by using the GeneFinder COVID-19 Plus RealAmp KIT assay (ELITech Group, Turin, Italy) according to the suggestions of the manufacturer. The Real-Time RT- PCR was carried out by using a Rotor-Gene-Q (Qiagen Hilden Germany) applying FAM fluorophore for the RdRp gene and JOE (ABI) fluorophore for the N gene. A positive test result was defined as a cycle threshold (Ct) ≤40 for individual targets.  *Quantitative RT‑PCR for*  200 ng of RNA was used to perform one step quantitative real-time reverse transcription PCR (TaqMan RNA-to-CT 1-Step Kit, Applied Biosystems, Foster City, CA, USA) at the appropriate annealing temperature for 40 cycles on a 7300 Real-Time PCR System (Applied Biosystems, Foster City, CA, USA). Data analysis was performed as relative quantification in relation to the expression of the housekeeping gene hypoxanthine-guanine phosphoribosyltransferase (HPRT) as internal standard. Specific primers and probes were purchased from MWG Biotech (Ebersberg, Germany). Primers were: hIL1b-fwd GCACGAT GCACCTGTACGAT, hIL1b-rev AGACATCACCAAG CTTTTTTGCT, hIL1b-probe ACTGAAC TGCACGCTCCGGGACTC, hIL6-fwd GGTACATCCTCGACGGCATCT, hIL6-rev GTGCCTC TTTGCTGCTTTCAC, hIL6-probe TGTTACTCTTGTTACATGTCTCCTTTCTCAGGGCT.  **Supplementary Table 1**  Organ weights   \| Organ weights \| Patient 1 (F 78a) \| Patient 2 (M 78a) \| Patient 3 (M 72a) \| Patient 4 (M 59a) \| \| --- \| --- \| --- \| --- \| --- \| \| Heart \| 527g \| 527g \| 411g \| 590g \| \| Lung right \| 815g \| 910g \| 1090g \| 1032g \| \| Lung left \| 659g \| 780g \| 815g \| 828g \| \| Liver \| 1350g \| 1705g \| 1940g \| 1730g \| \| Kidney right \| ---- \| 152g \| 175g \| 175g \| \| Kidney left \| ---- \| 145g \| 159g \| 165g \| \|  \|  \|  \|  \|  \|   **Supplementary Table 2**  Virus load found in different sites |  |  |  |  |  |  |  |  |
| --- | --- | --- | --- | --- | --- | --- | --- | --- | --- | --- | --- | --- | --- | --- | --- | --- | --- | --- | --- | --- | --- | --- | --- | --- | --- | --- | --- | --- | --- | --- | --- | --- | --- | --- | --- | --- | --- | --- | --- | --- | --- | --- | --- | --- | --- | --- | --- | --- |
| \| **Patient** \| **Organ** \| **Real time RT PCR (Ct value)** \| \| \| --- \| --- \| --- \| --- \| \| **RdRp gene** \| **N gene** \| \| 1 \| Heart \| n.d. \| n.d. \| \|  \| Lung \| 32.62 \| 23.74 \| \|  \| Liver \| n.d \| n.d. \| \| 2 \| Heart \| n.d. \| n.d. \| \|  \| Lung \| 38.2 \| 31.19 \| \|  \| Liver \| n.d. \| n.d. \| \| 3 \| Heart \| n.d. \| n.d. \| \|  \| Lung \| 27.48 \| 24.1 \| \|  \| Liver \| n.d. \| n.d. \| \| 4 \| Heart \| n.d. \| n.d. \| \|  \| Lung \| 40,6 \| 38,46 \| \|  \| Liver \| n.d. \| n.d. \| |  |  |  |  |  |  |  |  |
|  |  |  |  |  |  |  |  |  |
| n.d.= not detected |  |  |  |  |  |  |  |  |

|  |  |  |  |  |  |  | tod klinisch |
| --- | --- | --- | --- | --- | --- | --- | --- |

**Supplementary Figure 1**

Laboratory data patient 2

**Supplementary figure 2**

Patient 2

Pneumocyte type 1 containing virus particles


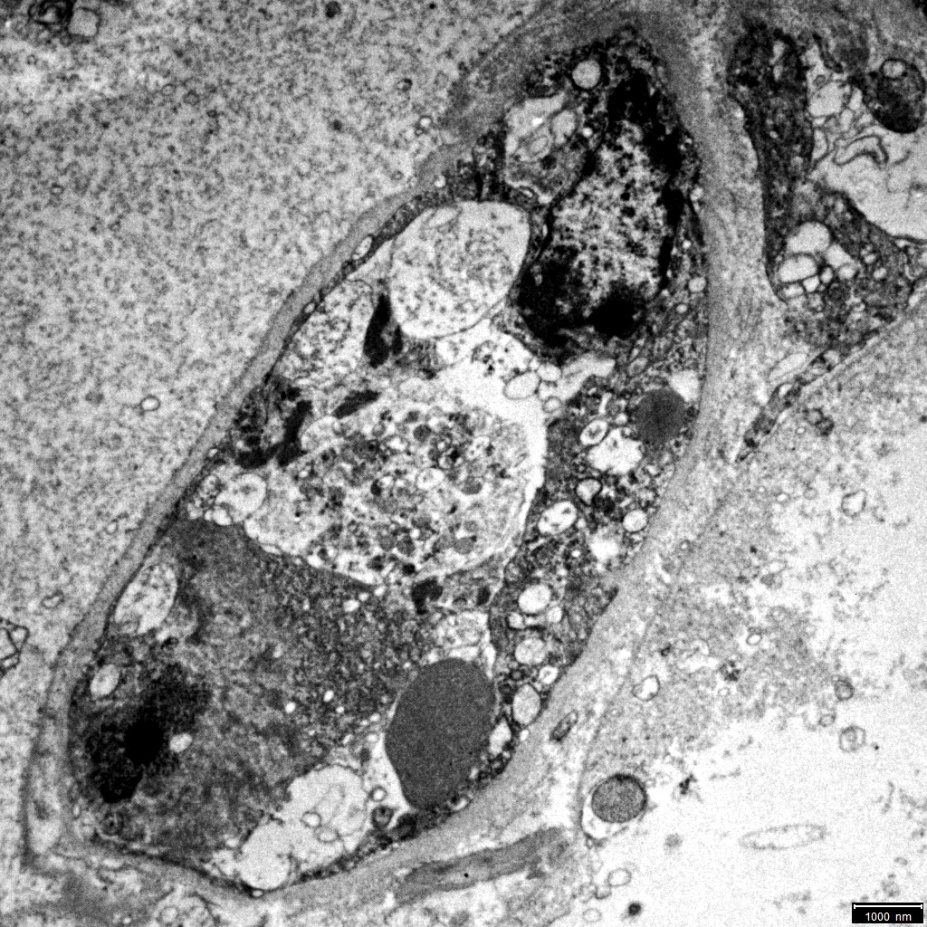

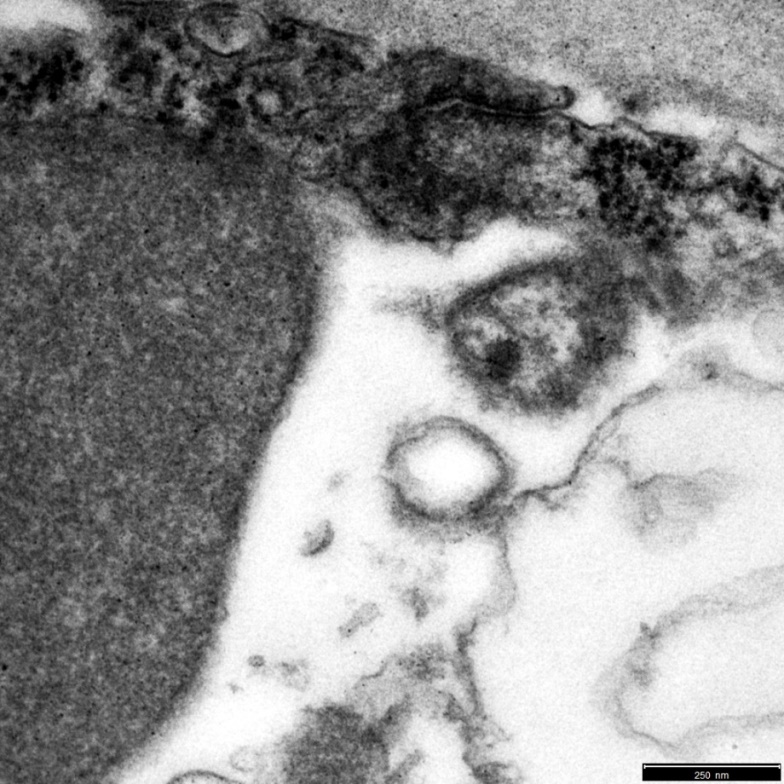

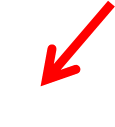
Insert: scale bar: 100 nm
